# Editorial: Emotions in digital contexts during the COVID-19 pandemic

**DOI:** 10.3389/fpsyg.2023.1231258

**Published:** 2023-07-03

**Authors:** Simone Belli, Juan C. Aceros, Angel Barrasa, Clara Selva, Barbara Sini, Antonietta Curci

**Affiliations:** ^1^Department of Social Anthropology and Social Psychology, Complutense University of Madrid and TRANSOC Institute, Madrid, Spain; ^2^Industrial University of Santander, Bucaramanga, Colombia; ^3^University of Zaragoza, Zaragoza, Aragon, Spain; ^4^Faculty of Psychology and Education Sciences, Open University of Catalonia, Barcelona, Catalonia, Spain; ^5^Department of Psychology, University of Turin, Turin, Piedmont, Italy; ^6^University of Bari Aldo Moro, Bari, Italy

**Keywords:** emotions, COVID-19, digital context, interaction, social connectedness

The ongoing COVID-19 pandemic has brought to the forefront the profound impact that digital contexts have on our emotional experiences. As social distancing measures have forced people to rely on virtual interactions to maintain social connections, the use of digital technologies has become more widespread than ever before. While these digital contexts can be a source of negative emotions, such as feelings of loneliness and isolation, they can also provide support and comfort during these difficult times. As a result, understanding the role of digital environments in shaping emotional experiences has become increasingly important. The Research Topic that we have collected showcases different studies that address this critical issue, providing a deep insight into how emotions manifest in digital environments. With this collection of research papers, we aim to contribute to the development of new psychological, educational, and technological strategies that promote positive emotional experiences and support wellbeing in both the short and long term.

Thanks to researchers from different parts of the world, this Research Topic is composed of eight articles divided into several sections such as Original Research, Method, Review, and General Comment.

In Kulke et al. present a cross-sectional observational study on the effects of sudden societal changes during the pandemic on mood and emotion recognition from eyes in both adults and adolescents. They found that mood was positively related to face-to-face but not virtual interactions in adults, and that virtual interactions were associated with negative mood in adolescents. This suggests that contact restrictions leading to decreased face-to-face interactions compared to virtual ones may be related to negative mood, understanding that prolonged exposure to people wearing masks during the pandemic could be related to increased sensitivity to subtle visual cues from others. Interestingly, the authors observed an improvement in the ability to recognize emotions from eyes during the pandemic compared to before, for both age groups. However, this improvement was only significant for females in the adult group, who were generally better at recognizing emotions from eyes. Overall, our results suggest that COVID-19 related contact restrictions may have impacted both adults and adolescents, particularly during this sensitive social development period.

In Zhao et al. demonstrate that the relationship between emotion and blended learning (BL) behavior supports the learner's wellbeing and an effective BL context. The results showed that students had an overall positive emotional experience with ups and downs, and face-to-face classes had more intense emotions than online learning, which had instead a more relaxed atmosphere. The study identified 11 influencing factors of academic emotions unique to blended learning, such as the degree of difficulty and interaction. These findings help to form a picture of students' academic emotions in the BL context. It also provides a practical reference for designing BL courses to inspire emotions that lead to effective and deep learning. According to the authors, future research can expand to other disciplines and use new learning analytic technologies for a more comprehensive picture of learners' emotional profiles.

In Mangiulli et al. present a study on fake news that examines whether people can form false memories of fake news related to COVID-19. In two different experiments, the authors demonstrated that false memories can occur after exposure to false news about COVID-19, and that government and social media interventions are needed to promote people's ability to discriminate between true and false news related to COVID-19. The experiments found that a significant percentage of people fell prey to COVID-19 fake news, particularly those related to negative effects of vaccination and pandemic tracking apps. The study also found that people with conspiracy beliefs were more likely to report false memories, irrespective of its conspiratorial content. The experiments identified several variables that could predict false memory formation, including wellbeing, virus-related anxiety, and analytic thinking. The findings suggest the importance of critical thinking abilities in resisting fake news and misinformation.

In Mejia et al. focus on media misinformation, which has caused fear and concern in Latin Americans. The results of the study – comparing twelve Latin American countries – showed that social networks and television were perceived as exaggerating the magnitude of events and generating fear, while physicians and health professionals were seen as provoking less fear. Furthermore, the study revealed Peru as the most affected country of all. The multivariate model shows that a higher level of exaggeration transmitted by the media perception was associated with being older and having severe or more serious anxiety or stress symptoms while a greater perception of fear and exaggeration of the information provided by professionals was associated with suffering from depression. Overall, the study points to an association between some mental pathologies and the perception that the media exaggerate the impact of COVID-related information.

In Zhang et al. present a study that establishes a detailed emotion classification model for annotating the emotions of 32,698 Sina Weibo posts related to COVID-19 prevention. The findings suggest that Chinese attitudes toward epidemic prevention and control are positive and optimistic; however, a notable proportion of fear and disgust is also recorded. The prevalent negative emotions were strongly linked to various concerning topics, such as the continued spread of the epidemic, inadequate management of contagious individuals, failure to implement effective prevention and control measures, as well as the occurrence of domestic and international outbreaks. This study is expected to help public health administrators in comprehending the prevailing public sentiments and promptly evaluating policies. Specifically, the identification of primary negative emotions and their associated topics can offer valuable insights into public concerns, thereby facilitating necessary policy adjustments and the implementation of more effective prevention and control measures of the COVID-19 epidemic.

In Martí et al. present a review on the teaching-learning process of reading and writing in the psycho-emotional and socio-psychological development of school-age children in confinement due to the COVID-19 pandemic. The results show the scarce presence of empirical studies on the subject. For the identification of recommendations in the development of face-to-face learning in the post-COVID era, it has been possible to identify some ideas of interest for future curricular designs in primary school students who are immersed in learning to read and write. According to the authors, addressing potential digital gaps in the student population is crucial, especially in ordinary times when socioeconomic disparities may exacerbate existing inequalities. For this purpose, the implementation of extracurricular programs or projects aimed at reducing these gaps could be highly beneficial. Such initiatives would provide students with access to essential digital tools and resources, enabling them to acquire crucial competencies that are increasingly relevant in today's digital era.

In Flores-Plata et al. present a commentary based on the article by Saladino et al. (2020), aiming to identifying new intervention perspectives based on digital devices for mental health. The purpose of the Commentary is to broaden the discussion about the feasibility of telepsychology, its main benefits, and the importance of training generations of psychotherapists in this new technological context. The authors suggests that in Latin American countries, the implementation of telepsychology services can significantly enhance mental and physical wellbeing by facilitating access to mental healthcare services. Moreover, designing and developing interventions tailored to the specific needs and characteristics of patients can further optimize the effectiveness of telepsychology services.

In Liu et al. introduce the Highly Dynamic and Reusable Picture-based Scale (HDRPS) to assess the individual's emotional experiences. The HDRPS is a dynamic and reusable picture-based scale that can access subjects' affect without relying on semantic indices. The authors' contribution has three unique features, i.e. its reliance on a large material library, which can be randomly combined to generate test items on different topics; its use of cell phones and computers for measurement; its ease of use and quick operation. These features make HDRPS an effective tool for a continuous assessment of daily emotions without placing an additional burden on subjects.

In summary, the COVID-19 pandemic has significantly impacted how people live their emotional experiences, and digital contexts have become crucial in facilitating social connections and emotional regulation during interpersonal interactions. This Research Topic has explored the role of digital contexts in shaping emotional experiences during the COVID-19 pandemic. Through an interdisciplinary approach, we aim to gain a deeper understanding of how digital contexts can influence the emotional wellbeing of individuals in these unprecedented times. We hope that the findings of this collection of research papers will inform the development of evidence-based strategies to support emotional wellbeing in the context of a pandemic.

## Author contributions

All authors listed have made a substantial, direct, and intellectual contribution to the work and approved it for publication.
